# Is GAPDH a relevant housekeeping gene for normalisation in colorectal cancer experiments?

**DOI:** 10.1038/sj.bjc.6605851

**Published:** 2010-09-21

**Authors:** J Caradec, N Sirab, D Revaud, C Keumeugni, S Loric

**Affiliations:** 1 INSERM U955, team 7, Créteil 94000, France; 2 Paris Est University, Créteil 94000, France; 3 Henri Mondor University Hospital, Biochemistry and Genetics Department, Créteil 94000, France

**Sir,**

We have read with great interest the study by Kontos *et al* (2010) in *British Journal of Cancer* about the use of L-DOPA decarboxylase as a prognostic marker for colorectal adenocarcinoma.

To assess the expression level of L-DOPA decarboxylase transcripts, the authors have performed qRT-PCR using GAPDH as the dedicated housekeeping gene (HKG) to normalise their results. However, no results concerning the stability of GAPDH that has lead to its use as the best housekeeper in their model system were available.

We have recently shown on human cell lines that choosing an unstable internal control gene could generate dramatic misinterpretations (Caradec *et al*, 2010a). In our study, and in our conditions, GAPDH was one of the most variable HKG so far impairing its use as a relevant normaliser. As this gene is likely to be emblematical to normalise gene expression results, we have developed a specific qRT-PCR with calibration curve to specifically study GAPDH expression in different cell lines grown with various hypoxic conditions. Our results unambiguously showed that GAPDH expression varies according to oxygen tension. We have also analysed GAPDH variability comparing meta-analysis data from microarray experiments on human samples available online (https://www.oncomine.com/resource/login.html). Using Oncomine 4.3, a powerful tool allowing rapid gene expression comparison between healthy and/or tumour human samples, we report here that GAPDH variability differs largely from one study to another and more importantly may largely vary between patients in a given study (Figure 1). As, aside still unidentified factors, hypoxia can be considered as a major one to have a critical role in cancer development, especially in colorectal cancer (Baba *et al*, 2010), we would be interested in learning how Kontos *et al* have tested HKGs variability in their system and found GAPDH to be the most relevant.

Another unclear point concerns qRT-PCR *C*_t_ intervals between GAPDH and L-DOPA target gene amplification. Indeed, the study results of Kontos *et al.* showed a Δ*C*_t_ equal to 13 (*C*_t_ (GAPDH) 15, *C*_t_ (L-DOPA) 28), signifying that GAPDH transcripts are likely to be expressed 2^13^ (8200) times higher than those encoding L-DOPA. As we stressed this particular point very recently, discussing about the use of r18S as a normaliser (Caradec *et al*, 2010b), we would be very interested to know Kontos *et al.* opinion about the accuracy of such disproportion.

Definitely, the choice of a valid HKG set will determine the relevance of the results that will be further interpreted, and so it should be seriously considered.

## Figures and Tables

**Figure 1:**
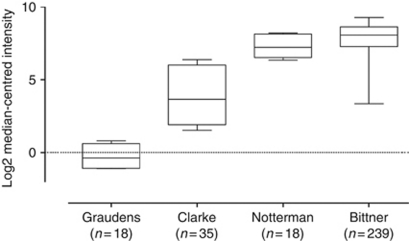
GAPDH fold change in different colon adenocarcinoma studies (Graudens *et al*, 2006; Clarke *et al*, 2003; Notterman *et al*, 2001 and Bittner, not published (International Genomics Consortium, *Expression Project for Oncology - Colon Samples,* http://www.ncbi.nlm.nih.gov/geo/query/acc.cgi?acc=GSE2109)). Zero on the bar chart scale represents no change, with each increment on the scale representing a two-fold difference. In Bittner study, it means that the observed 5.922 interval between maximal and minimal values corresponds to a 2^5.922^, i.e., 60 GAPDH fold change.
